# BDNF and IL-8, But Not UCHL-1 and IL-11, Are Markers of Brain Injury in Children Caused by Mild Head Trauma

**DOI:** 10.3390/brainsci10100665

**Published:** 2020-09-24

**Authors:** Marzena Tylicka, Ewa Matuszczak, Adam Hermanowicz, Wojciech Dębek, Maria Karpińska, Joanna Kamińska, Olga Martyna Koper-Lenkiewicz

**Affiliations:** 1 Department of Biophysics, Medical University of Białystok, Mickiewicza 2a, 15-089 Białystok, Poland; karpm@umb.edu.pl; 2 Department of Pediatric Surgery and Urology, Medical University of Białystok, Waszyngtona 17, 15-274 Białystok, Poland; ewamat@tlen.pl (E.M.); ahermanowicz@wp.pl (A.H.); wdebek@umwb.edu.pl (W.D.); 3 Department of Clinical Laboratory Diagnostics, Medical University of Białystok, Waszyngtona 15A, 15-269 Białystok, Poland; joanna.kaminska@umb.edu.pl

**Keywords:** mild head trauma, brain-derived neurotrophic factor (BDNF), interleukin-8 (IL-8), interleukin-11 (IL-11), ubiquitin C-terminal hydrolase L1 (UCHL-1)

## Abstract

The aim of the study was to check whether the plasma levels of brain-derived neurotrophic factor (BDNF), interleukin-8 (IL-8), interleukin-11 (IL-11) and ubiquitin C-terminal hydrolase L1 (UCHL-1) change in children with mild head trauma (*N* = 29) compared to controls (*N* = 13). Protein concentration in children with mild head trauma (12 children with mild concussion without loss of consciousness and 17 children with severe concussion and loss of consciousness) and the control group were measured by means of the Enzyme-Linked Immunosorbent Assay (ELISA) method. IL-8 and BDNF concentration was statistically higher in the group of children with mild head trauma (9.89 pg/mL and 2798.00 pg/mL, respectively) compared to the control group (7.52 pg/mL and 1163.20 pg/mL, respectively). BDNF concentration was significantly higher in children with severe concussion and loss of consciousness (3826.00 pg/mL) than in the control group. None of the tested proteins differed significantly between children with mild concussion without loss of consciousness and children with severe concussion and loss of consciousness. BDNF and IL-8 may be sensitive markers of brain response to mild head trauma in children. The lack of statistical differences for BDNF and IL-8 between children with mild or severe concussion could indicate that their elevated levels may not result from significant structural brain damage but rather reflect a functional disturbance.

## 1. Introduction

Head trauma is one of the most common types of injury in children and adolescents [1,2,3]. The World Health Organization defines head injury as any injury to the scalp, skull or brain [4]. Severe head injury causes shearing of the long axons and rapid acceleration-deceleration of brain substance which in consequence leads to a diffuse axonal injury and alterations in consciousness. On the contrary, in the case of mild head injury, these changes are less severe and only connected with microscopic diffuse axonal injury. However, animal studies show that even minor blows to the head can lead to neural injury. In humans, mild head trauma also causes neural damage [5]. In some cases mild head injury may result in concussion, which is defined as a reversible neurological dysfunction in the absence of gross brain lesions [1,6,7]. Concussion is one of the least understood injuries. Symptoms of a concussion vary depending on the severity of the head injury and whether the person injured is a child or adult. Mild concussion often occurs without loss of consciousness, causing confusion and problems with memory, speech, vision, or balance [8,9]. Severe concussion occurs with a loss of consciousness, which varies in duration and depth. Returning to everyday activities after a mild concussion may be permissible after a day or two. After a severe concussion, the patient usually needs a few weeks of recovery time [8,9].

The culmination of neuronal damage ultimately triggers an inflammatory response. Inflammatory response to head injury is mediated by dynamic interactions between multiple cell types. In particular, glial cells are key to the initiation and the pathological prolongation of neuroinflammation [10]. Under normal condition these cells are suppressed by peptide signaling from healthy neurons, but in response to injury microglia become activated and release inflammation-promoting mediators such as complement factors, cytokines, chemokines, proteolytic enzymes, reactive oxygen and nitrogen species [10]. There is some evidence that the concentration of inflammatory proteins, such as IL-6, IL-8, and IL-10, is increased following neurotrauma [11]. IL-8 appears to peak early following brain trauma and its expression induces chemotaxis and phagocytosis of neutrophils, attracting them to the site of the neural damage in order to clean up the debris resulting from the injury [12,13]. A previous study also indicates that IL-11, which is a neuropoietic cytokine, may be involved in the control of neuronal, glial and immune response to injury. Neurokines are central to many brain processes and may have signaling functions in nervous system development [14]. Cytokine response to head injury may have also an impact on neurogenesis via the modulation of neurotropic factors biosynthesis, including brain-derived neurotrophic factor (BDNF) and nerve growth factor (NGF) [15,16,17,18]. NGF binds to the tropomyosin receptor kinase A (TrkA) receptor, which activates ERK 1/2 and Akt pathways and stimulates the transcription of pro-survival genes and inhibits cell apoptosis. BDNF binds to the TrkB receptor [19,20]. Both NGF and BDNF bind to the non-specific neurotrophin (NT) receptor p75 [18]. A previous study presented that children with traumatic brain injury showed statistically higher BDNF than NGF levels, both in the cerebrospinal fluid and plasma [16]. BDNF plays a pivotal role in the survival of neurons, regulation of neurite outgrowth and maintenance of synaptic connectivity of the central nervous system [19]. Studies on animal models show that exogenous administration of neurotropic factors reduces or even prevents neurological deficits and cell death after brain injury [17].

Recent studies have concentrated on the ubiquitin carboxyl-terminal hydrolase isoenzyme L1 (UCHL-1), a deubiquitinase that is highly expressed in neurons, as a possible marker of brain injury [21,22]. The neuronal protein UCHL-1 is involved in the addition and removal of ubiquitin proteins flagged for metabolism [11].

Because BDNF, IL-8, IL-11 and UCHL-1 response to mild head injury with concussion has not been widely studied so far, as it occurs mostly in cases of traumatic brain injury, the aim of the current study was to test whether the levels of the abovementioned proteins are changed in children with mild concussion without loss of consciousness (LOC) compared to children with severe concussion and LOC. Therefore, circulating IL-8, IL-11, BDNF and UCHL-1 concentrations were measured in the plasma of these patients.

## 2. Material and Methods

### 2.1. Patients

The study population comprised 29 children admitted to the Pediatric Surgery Department of the Medical University of Bialystok due to a mild head trauma between 2018 and 2020. Inclusion criteria were female or male patients aged between 7 and 17. Those patients whose parents gave informed consent for both clinical and biochemical follow-up were admitted into the study. Blood samples from children with mild head trauma were collected within 2–6 h after the injury had occurred.

The study group was divided by taking into consideration the grade of concussion resulting from mild head trauma: children with mild concussion without loss of consciousness (LOC) (*N* = 12), and children with severe concussion with LOC (*N* = 17). Falls and blows were the most common mechanisms of mild head trauma among both subgroups of children. The diagnosis of mild head trauma with concussion was based upon the presence of some the following symptoms: headache, vomiting, dizziness, problems with balance, fatigue, sensitivity to light or noise, slowed thinking, problems with memory, concentration or focus, problems with sleep, Glasgow Coma Scale (GCS) score 13–15 and normal head computed tomography (CT).

Exclusion criteria were GCS scores below 13, abnormal head CT, hospital admission later than 6 h after trauma, pre-existing infections or diseases which required long-term medication.

Thirteen healthy children, aged matched to the study group, admitted to the Department of Pediatric Surgery and Urology for planned inguinal hernia repairs and whose parents gave informed consent for both clinical and biochemical follow-up served as the control group. 

Approval for this study was obtained from Bioethics Committee of Medical University of Białystok (No: R-I-002/229/2018). Procedures were in accordance with the ethical standards set by the Declaration of Helsinki given by World Medical Association.

### 2.2. Plasma BDNF, IL-8, IL-11, UCHL-1 Concentration Measurement

Blood samples from children with mild head trauma and the control group were collected in test tubes with EDTA-K (S-Monovette^®^ 1.2 mL K3E, SARSTEDT, Nümbrecht, Germany). After the centrifugation (20 min at 1000× *g* force), plasma samples were aliquoted and stored at −80 °C until further analysis.

BDNF concentration was measured using an ELISA Quantikine^®^ Human Free BDNF Immunoassay kit (Catalog number: DBD00; R&D Systems Europe Ltd., Abingdon, UK) according to the manufacturer’s instructions. Plasma samples were diluted 20-fold prior to analysis (10 µL of sample and 190 µL of Calibrator Diluent RD6P). The manufacturer of the assay kit referred to the intra-assay coefficient of variation (CV%) as 4.7% at BDNF mean concentration of 334 pg/mL, SD = 15.6 pg/mL.

IL-8 concentration was measured using an ELISA Quantikine^®^ Human CXCL-8/IL-8 Immunoassay kit (Catalog number: D8000C; R&D Systems Europe Ltd., Abingdon, UK) according to the manufacturer’s instructions. Plasma samples were not diluted prior to analysis. The manufacturer of the assay kit referred to the intra-assay coefficient of variation (CV%) as 5.6% at IL-8 mean concentration of 168 pg/mL, SD = 9.4 pg/mL.

IL-11 concentration was measured using an ELISA Quantikine^®^ Human IL-11 Immunoassay kit (Catalog number: D1100; R&D Systems Europe Ltd., Abingdon, UK) according to the manufacturer’s instructions. Plasma samples were not diluted prior to analysis. The manufacturer of the assay kit referred to the intra-assay coefficient of variation (CV%) as 2.4% at IL-11 mean concentration of 70.7 pg/mL, SD = 1.7 pg/mL.

UCHL-1 concentration was measured using an ELISA Ubiquitin Carboxyl Terminal Hydrolase L1 (UCHL1) kit (Catalog number: SEG945Hu; Cloud-Clone Corp., Katy, TX, USA) according to the manufacturer’s instructions. Plasma samples were not diluted prior to analysis. The manufacturer of the assay kit referred to the intra-assay coefficient of variation (CV%) as <10%.

### 2.3. Data Analysis

Statistical analysis was performed using the STATISTICA PL release 12.5 Program. All the results are presented as median with 25th and 75th percentiles. The results were analyzed by the Mann-Whitney U test (two-sided nature) because the tested parameters did not pass the normality test. The data was previously tested for normality using the Shapiro-Wilk test. Differences between two independent groups were considered significant with a value of *p* < 0.05.

Correlations were studied by using the Spearman correlation test. Differences were considered significant with a value of *p* < 0.05.

## 3. Results

**Figure 1:**
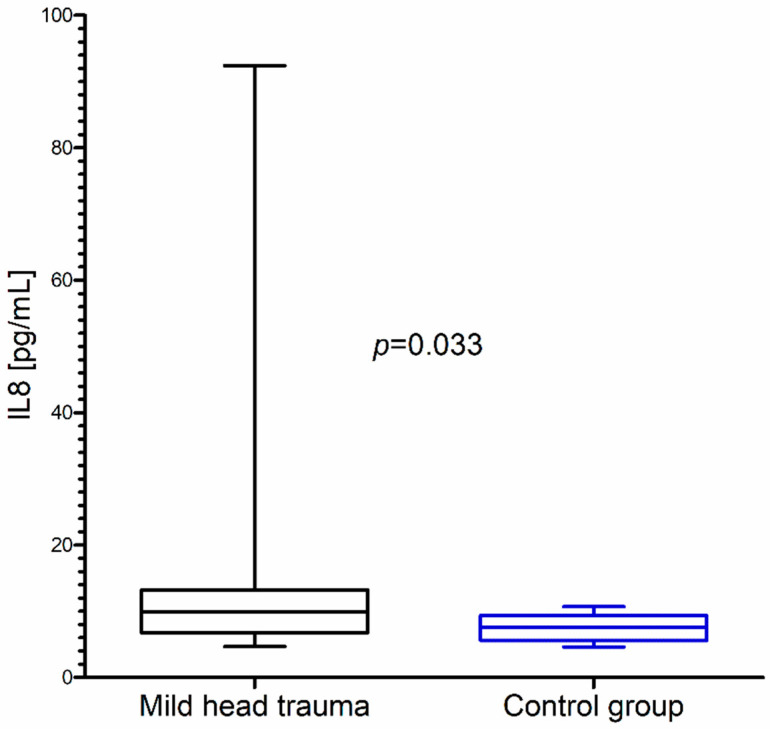
Interleukin-8 (IL-8) concentration in the total group of children with mild head trauma compared to the control group.

**Figure 2:**
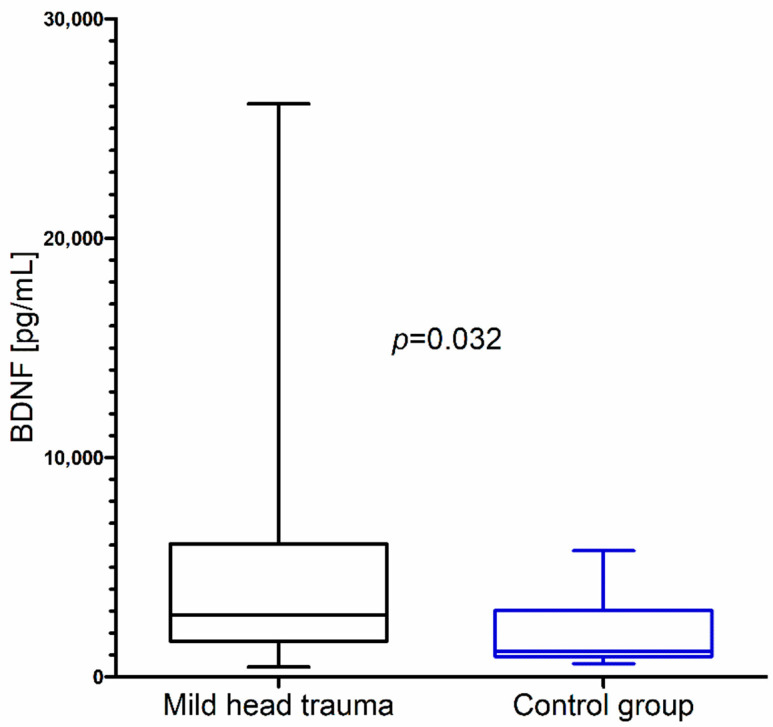
Brain-derived neurotrophic factor (BDNF) concentration in the total group of children with mild head trauma compared to the control group.

Within the whole group of children with mild head trauma, a statistically significant increase of IL-8 and BDNF concentration in comparison to the control group was observed (Figure 1 and Figure 2) (Table 1).

**Figure 3:**
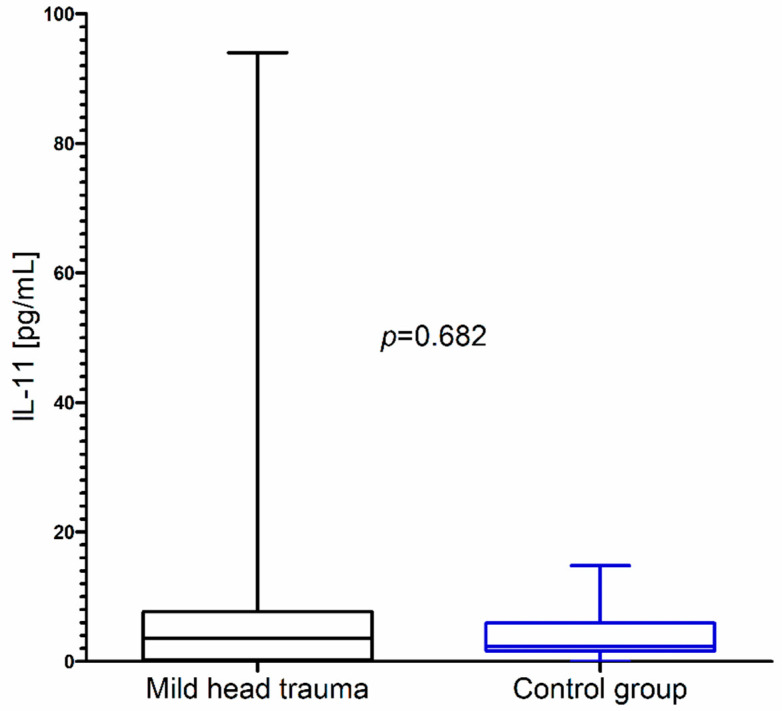
IL-11 concentration in the total group of children with mild head trauma compared to the control group.

In contrast to these results, we did not observe significant changes in IL-11 concentration as a consequence of mild head trauma (Figure 3) (Table 1).

UCHL-1 levels in both children with mild head trauma and the control group were below the detection limit of the assay kit.

**Figure 4:**
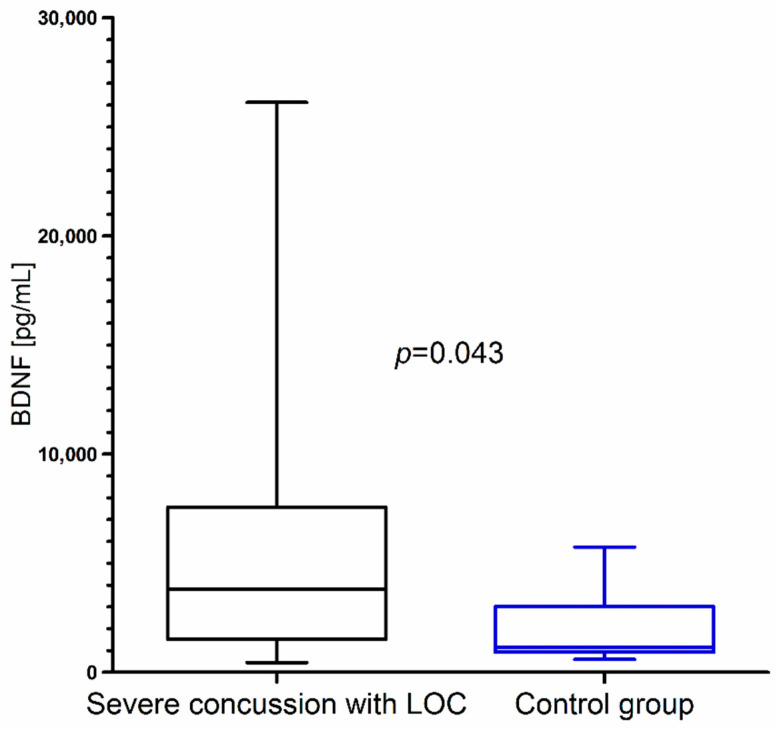
BDNF concentration in children with severe concussion with loss of consciousness (LOC) resulting from mild head trauma compared to the control group.

Closer inspection showed that BDNF concentration was significantly higher in children with severe concussion with LOC than in the control group (Figure 4). We did not observe statistical differences in BDNF concentration between children with mild concussion without LOC and the control group (*p* = 0.682).

Comparing BDNF concentration between children with mild and severe concussion, we found that BDNF concentration was higher after severe concussion with LOC, but the difference between groups was not statistically significant. Furthermore, IL-8 concentration did not differ between both subgroups of children with mild head trauma (Table 2).

In the next step we correlated the tested molecules with each other for the whole group of children with mild head trauma, however we did not observe any statistical correlation coefficients (*p* > 0.05).

## 4. Discussion

The culmination of neuronal damage triggers an inflammatory response, which in consequence can be beneficial and confer neuroprotection of the central nervous system. It was found that cells from the immune system produce and secrete a variety of neurotrophins such as: NGF, BDNF, NT-3 and NT-4/5 [23]. The neuroinflammatory cascade is mediated by the release of cytokines and chemokines, therefore microglia are the primary source of inflammatory mediators in the brain [24]. In response to the head trauma, the balance of the two cellular systems, anti-inflammatory (encompassing neuroprotective neurotrophins and other chemical mediator secretions) and proinflammatory (encompassing neurotoxic tumor necrosis factor (TNF), free radicals, and certain cytokine secretions), is significant in the maintenance of homeostasis [23].

Among many proinflammatory mediators, chemokine IL-8 has been found to induce neurotrophin production after brain injury [25]. Moreover this chemokine induces chemotaxis and phagocytosis of neutrophils, attracting them to the site of neural damage [12,26]. Several studies demonstrate acute and persistent IL-8 level increase following severe traumatic brain injury [27,28,29]. Whalen et al. [27] observed that cerebrospinal fluid (CSF) IL-8 is markedly increased in children with severe traumatic brain injury. They suggest that this chemokine may play an important role in both injury and regenerative processes after traumatic brain injury and its high concentration correlates with mortality [27]. Maier et al. [28] demonstrated that CSF and plasma IL-8 levels were significantly increased early after trauma compared to the baseline levels [28]. Gołąbek-Dropiewska et al. [29] found significant changes in IL-8 levels in patients after trauma compared to healthy subjects, which also confirm the significance of IL-8 evaluation after head trauma. A current study has also indicated that there was a statistically significant difference between IL-8 concentration in children after mild head trauma and the control group, which also supported previous findings that IL-8 increases in response to head trauma. However, we did not find significant differences between children with mild concussion without LOC and severe concussion with LOC. Similar IL-8 median values in both mild head injury subgroups may result from the fact that the trauma per se significantly affects the course of inflammatory response. On the other hand, neuropathological changes manifested in the form of concussion after head injury may result from functional disturbance rather than more severe structural brain damage [29,30,31], which could also account for the lack of difference in IL-8 levels. This postulate was confirmed by Schimmel et al. [32], who indicated that neuroinflammation in response to traumatic brain injury increases neural cell death by interfering with endogenous repair mechanisms and acts through chemokines, cytokines and other inflammatory molecules. Activation of an inflammatory response occurs due to repair of damaged cells and protects the brain from pathogens [32].

IL-11 as a neuropoietic cytokine is well known for its role in the control of glial, neuronal and immune response to brain injury. According to Bauer et al. [14], neuropoietic cytokines, including IL-11, have signaling functions in response to brain damage. Ito et al. [33] indicate that these kind of cytokines are upregulated by nerve injuries. In our study, we observed slightly elevated plasma IL-11 concentration in children after head trauma compared to control individuals, however the obtained difference was not significant. Thus, our observation may in some respects confirm the study of Ito et al. [33], as our study population included only those individuals with mild head injury, which was unlikely to cause severe brain damage.

Neuronal cells require neurotrophins, including BDNF and NGF, to promote recovery from damage caused by traumatic injuries [16,34,35,36]. Neurotrophin concentration is significantly different in cerebrospinal fluid and plasma of patients with hypoxia, as well as seizures and neuroimmunological diseases [16]. However the role of BDNF and NGF in human traumatic brain injury has not been widely studied so far [37,38,39]. The only studies encompassing children are reported by Chiaretti et al. [16,17]. The authors show that BDNF and NGF levels are significantly higher in the cerebrospinal fluid and plasma of children with severe traumatic brain injury, compared to the control group [16,17]. Moreover, the authors measured cerebrospinal fluid and plasma BDNF and NGF concentration both 2 and 24 h after head injury and analyzed them depending on the clinical outcome. Based on the obtained results, Chiaretti et al. [17] postulated that BDNF represents an early marker of brain injury, while increased NGF concentration in the CSF is indicative of a good outcome. The authors went even further to hypothesize that NGF may have a role in the treatment of children with severe head injury.

Our observations demonstrated that even in the case of patients with mild head trauma, the growth of plasma BDNF concentration was statistically significant in comparison to the control group. The current study is also one of the first to show that plasma BDNF concentration is higher in the group of children with severe concussion with LOC than in those with mild concussion without LOC, although this difference was not statistically significant (*p* > 0.05). Singh et al. [34] postulated that BDNF plays an important role as a neurotransmitter modulator and is an active participant in neuronal plasticity, which is essential for learning and memory. This fact might explain why we observed a tendency for BDNF to be higher in children with mild head trauma with LOC compared to those without LOC.

Some studies report the protein ubiquitin C-terminal hydrolase L1 (UCHL-1) as a novel potential biomarker of brain injury [40,41,42]. Our previous studies show that in children, UCHL-1 is overexpressed in cases of cryptorchidism, acute appendicitis and thermal injury [43,44,45,46]. Brophy et al. [40] as well as Mondello et al. [47] demonstrated increased serum UCHL-1 concentration in adults after traumatic brain injury. Mondello et al. [47] postulated that UCHL-1 is detectable in the blood after an injury, relates to the magnitude of the injury and could be an early predictor of mortality. Increased serum levels of UCHL-1 [42] also correlate with a worse outcome for children after traumatic brain injury. Berger et al. [42] concluded that this protein may be useful in predicting the outcome after moderate and severe pediatric traumatic brain injury. The authors observe that the lack of UCHL-1 increase in children with mild injury could be associated with the absence of neuronal and axonal injury or with a lack of analysis sensitivity to minor damage [42]. Our results are in line with these observations, as we noticed that in all children with mild head injury with severe and mild concussion, plasma UCHL-1 concentrations were under the detection limit.

The limitation of the study is the small number of children analyzed, as the number of observations has an impact on whether the obtained result is statistically significant or not [48]. However, in our study, despite the small number of cases, we obtained statistically significant differences. By way of justification for the small number of cases included in the study, we would like to explain that blood samples were collected only from those children whose parents gave their informed written consent.

Another study limitation is the lack of evaluation of NGF concentration and neurotrophin receptor (Trk-A/Trk-B and p75) changes to find out if they are up- or downregulated. But our current study was limited by the material obtained from pediatric patients, as we received permission from the local Bioethics Committee to collect only 1.2 mL of the whole blood to obtain plasma. Taking into account the fact that neurotrophin receptor expression changes in traumatic brain injury patients have not been studied so far, this aspect warrants further development.

## 5. Conclusions

BDNF and IL-8 may be sensitive markers of brain response to mild head trauma in children. The lack of statistical differences for BDNF and IL-8 between children with severe concussion with LOC compared to those with mild concussion without LOC may indicate that the occurrence of a concussion does not result from significant structural brain damage but rather reflects a functional disturbance. UCHL-1 and IL-11 are not useful markers in the case of mild head trauma because their concentrations did not change in comparison to the control group.

## Figures and Tables

**Table 1:** IL-8, BDNF and interleukin-11 (IL-11) concentration in children with mild head trauma compared to the control group.

	IL-8 [pg/mL]		BDNF [pg/mL]		IL-11 [pg/mL]	
	Mild Head Trauma (*N* = 29)	Control Group (*N* = 13)	Mild Head Trauma (*N* = 29)	Control Group (*N* = 13)	Mild Head Trauma (*N* = 29)	Control Group (*N* = 13)
Median	9.89	7.52	2798.00	1163.20	3.54	2.27
Percentiles (25–75%)	6.82–13.24	5.97–8.94	1641.70–5856.00	937.00–2892.00	0.23–7.35	2.04–4.72
*p*-value *	*p* = 0.033		*p* = 0.032		*p* = 0.682	

* A *p*-value < 0.05 is considered to show a significant difference between groups.

**Table 2:** BDNF and IL-8 concentration in children with mild head trauma: mild concussion without LOC vs. severe concussion with LOC.

	IL-8 [pg/mL]		BDNF [pg/mL]	
	Mild Concussion without LOC (*N* = 12)	Severe Concussion with LOC (*N* = 17)	Mild Concussion without LOC (*N* = 12)	Severe Concussion with LOC (*N* = 17)
Median	10.79	9.41	2650.00	3826.00
Percentiles (25–75%)	8.74–19.83	6.74–11.92	1623.40–5340.00	1660.00–6258.00
*p*-value *	*p* = 0.223		*p* = 0.605	

* A *p*-value < 0.05 is considered to show a significant difference between groups.

## Abbreviations

BDNF	brain-derived neurotrophic factor
CSF	cerebrospinal fluid
CT	computed tomography
EDTA-K	potassium ethylenediaminetetraacetic acid
ELISA	Enzyme-Linked Immunosorbent Assay
GCS	Glasgow Coma Scale
IL	interleukin
NGF	nerve growth factor
NT	neurotrophin
TNF	tumor necrosis factor
Trk	tropomyosin receptor kinase
UCHL-1	ubiquitin carboxyl-terminal hydrolase isoenzyme L1
